# Pause-melting misalignment: a novel model for the birth and motif indel of tandem repeats in the mitochondrial genome

**DOI:** 10.1186/1471-2164-14-103

**Published:** 2013-02-15

**Authors:** Wei Shi, Xiao-Yu Kong, Zhong-Ming Wang, Shan-Shan Yu, Hai-Xia Chen, Elizabeth A De Stasio

**Affiliations:** 1 Key Laboratory of Marine Bio-resource Sustainable Utilization (LMB), South China Sea Institute of Oceanology, Chinese Academy of Sciences, 164 West Xingang Road, Guangzhou 510301, Republic of China; 2 Department of Biological and Environmental Sciences, University of Gothenburg, PO Box 463, SE-405 30, Gothenburg, Sweden; 3 Biology Department, Lawrence University, 711 E. Boldt Way, 54911, Appleton, WI, USA

## Abstract

**Background:**

Tandem repeats (TRs) in the mitochondrial (mt) genome control region have been documented in a wide variety of vertebrate species. The mechanism by which repeated tracts originate and undergo duplication and deletion, however, remains unclear.

**Results:**

We analyzed DNA sequences of mt genome TRs (mtTRs) in the ridged-eye flounder (*Pleuronichthys cornutus*), and characterized DNA sequences of mtTRs from other vertebrates using the data available in GenBank. Tandem repeats are concentrated in the control regions; however, we found approximately 16.6% of the TRs elsewhere in the mt genome. The flounder mtTRs possess three motif types with hypervariable characteristics at the 3′ end of the control region (CR).

**Conclusion:**

Based on our analysis of this larger dataset of mtTR sequences, we propose a novel model of Pause Melting Misalignment (PMM) to describe the birth and motif indel of tandem repeats. PMM is activated during a pause event in mitochondrial replication in which a dynamic competition between the nascent (N) heavy strand and the displaced (D) heavy strand may lead to the melting of the N-strand from the template (T) light strand. When mispairing occurs during rebinding of the N-strand, one or several motifs can be inserted or deleted in both strands during the next round of mt-replication or repair. This model can explain the characteristics of TRs in available vertebrate mt genomes.

## Background

The mitochondrial (mt) genome appears to evolve faster than the single-copy fraction of the nuclear genome by approximately a factor of ten [1]. The portion of the mt genome that evolves most rapidly is the control region (CR), or the D-loop region, presumably due to lower selection pressure [1-3]. Within the control region of vertebrate mt genomes, tandem repeats (TRs) located at the CR termini (the 3′- or 5′- end) evolve most rapidly, with extensive variations in motif size and copy number resulting from motif insertions and deletions (indels) in TR tracts [4-7].

To date, several hypotheses have been proposed to explain the birth and indel of mt genome tandem repeats (mtTRs). Two related theories explaining the mechanism by which motif indels are created in nuclear genomes have been applied to similar events in mt genomes. In one such theory, recombination, which has been confirmed in mt genomes [5,8-11], is responsible. In this case, double strand breakage and rejoining of DNA lead to the gain or loss of mtTR motifs. An alternative hypothesis, the Slipped-Strand Mispairing (SSM) model [12], not only accounts for microsatellite indels [13-15], but also for motif indels in mtTRs [6,16,17]. In its simplest form, the SSM model involves local denaturation and displacement of the strands of a DNA duplex, followed by mispairing of complementary bases at the site of an existing similar sequences or short tandem repeat. When followed by replication or repair, misalignment could lead to indels of one or several simple repeats.

A few models have been proposed to explain particular indel events in mt genomes [18-21], and subsequently have been adopted in some other cases [5,22-24]. For example, the Illegitimate Elongation Model (IEM) suggested by Buroker [18] involves a competitive equilibrium between the nascent (N) heavy strand and the displaced (D) heavy strand while the N-strand is arrested at a termination-associated sequence (TAS) in the 5′ end of the CR. This model suggests that frequent misalignment occurs prior to elongation in the repeat region and is facilitated by a stable secondary structure in the D-strand. This mechanism could result in both length and sequence heteroplasmy of TRs. Another influential model, the Improper Initiation Model (IIM), was proposed by Broughton and Dowling [19]. In this process, mt-replication begins at the upstream *tRNA*^*Phe*^ gene, an improper origin of mt-replication. If mt-replication is completed at the regular termination site, the sequence between *tRNA*^*Phe*^ and the normal origin of replication will be repeated on the nascent heavy strand. Under these circumstances, the repeated sequence will always be identical in length and position. Other mechanisms used to explain the origin of special motif indels include that of Taylor and Breden [20], who present a model for mini-satellite birth from non-contiguous repeats in the mt genome of the guppy, *Poecilia reticulate,* and that of Hayasaka [21], used to explain the motif indels of mtTRs involving conserved sequence blocks 2 and 3 (CSB2, CSB3) in the CR of the Japanese monkey, *Macaca fuscata*.

Each of these models possesses certain limitations. Previous studies have provided some evidence of recombination in mt genomes, but the incidence of mitochondrial DNA recombination is thought to be rare [7,25-27]. In fact, indels of repeat regions in mtDNA are too abundant to be explained solely by the recombination model. A prediction of the SSM model, due to its intra-helical nature, is that there should be an appreciable bias toward the duplication of short simple repeat units; therefore, the likelihood of a tandem repeat forming would decrease rapidly as the length of the repeat motif increases [20]. Consequently, this model is unlikely to explain the higher proportion of long motif repeats. Both IEM and IIM best explain the formation of motif indels only in a particular position of the CR.

How do tandem repeats form, and subsequently insert and delete in mt genomes, and why do they have high sequence and length heteroplasmy even within an individual? In order to address these questions, we analyzed the sequences of highly variable mtTRs in the ridged-eye flounder (*Pleuronichthys cornutus*), and explored the characteristics of vertebrate mtTRs using data from GenBank. We then developed a novel model for the birth and motif indel of TRs, in the hope of further understanding the evolutionary mechanism of mtTRs variability, and the evolution of mt genomes more generally.

## Methods

### The amplification of *P. cornutus* CRs

We used 20 individuals of *P. cornutus*, thirteen from Dongshan, East China Sea and seven from Qingdao, Yellow Sea. A portion of the epaxial musculature was excised from each fresh specimen, and immediately stored at −70°C. Total genomic DNA was extracted using an SQ Tissue DNA Kit (OMEGA, China) following the manufacturer’s protocol. Two primer pairs (L-DL: ACTCCCAAAGCCAGGATTCT, H-DL: GAGGGTGAGGTTTAACGGGGG; L-AP1: GCCTGTAGCTTTTTAGGTAT, H-AP2: AAGCATAACACTGAAGATG) were designed for amplification of *P. cornutus* control region sequences. The PCR was performed in a 25μl reaction volume containing 2.0 mM MgCl_2_, 0.4 mM dNTP, 0.5 μM of each primer, 1.0 U Taq polymerase (Takara, Dalian, China), 2.5 μl 10x Taq buffer, and approximately 50 ng DNA template. PCR cycling conditions included an initial denaturation at 95°C for 3 min, 35 cycles of a denaturation at 94°C for 45 s, an annealing step at 48°C for 45 s, and elongation at 72°C for 3 min with a final extension at 72°C for 5 min. The PCR products were detected in 1.0% agarose gels and purified using a Takara Agarose Gel DNA Purification Kit (Takara, China). Purified products were inserted into the PMD18-T vector (Takara, China), and then transformed into *E. coli* competent cells. The final cloned products were sequenced in both directions using the ABI 3730 genetic Analyzer. Primers used in sequencing were the same as those used for PCR, and new primers were designed for walking sequencing. The sequenced fragments were assembled using DAMBE v 5 [28] and BioEdit v 7.0.1 [29].

### GenBank database and TRs analysis

The sequences of 1726 vertebrate mt genomes were retrieved from the NCBI genome database in March, 2012 (http://www.ncbi.nlm.nih.gov/genome). Repeated sequences were identified using Tandem Repeats Finder v4.03 [30] with the following parameters: match +2, mismatch −7, indel −7. Only those repeats whose scores exceeded 50 were reported. Looser or stricter parameters were also tested in the same software packages, but these resulted in more paradoxical repeats and the loss of some repeats from the dataset.

The location of TRs in mt genomes from GenBank was manually checked, and TRs located in the CRs were classified by their location relative to the 3′ or 5′ end. Those TRs without a clear location in the CR were not used in the statistical analysis. Utilizing SPSS software, the distributions of motif lengths in the 3′ and 5′ ends and in protein coding genes were analyzed.

## Results and discussion

### The configurations of *P. cornutus* mtTRs

**Figure 1:**
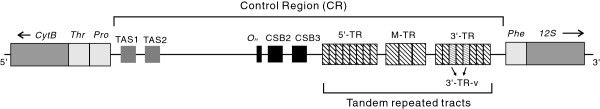
**An illustration of the location of three motif types in *P. cornutus *control region. ***CytB*: Cytochrome b gene; Thr, Pro, Phe: tRNA of Threonine, Proline, Phenylalanine; *12S*: Small subunit ribosomal DNA.

**Figure 2:**
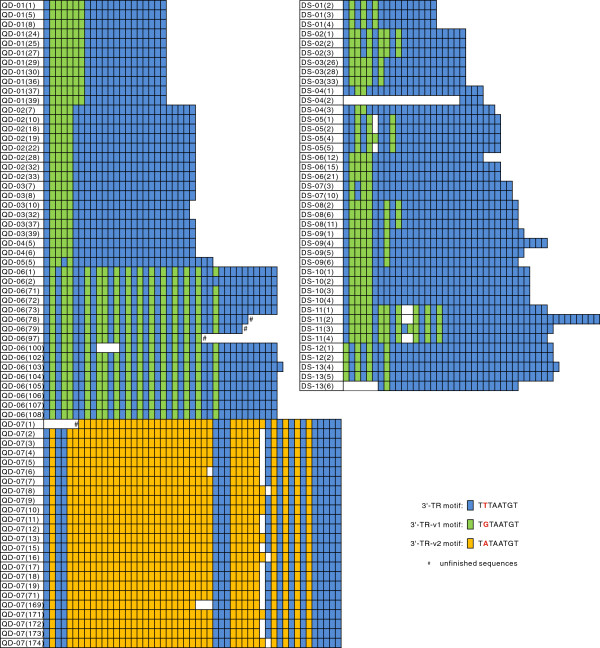
**Comparisons of 3′-TR tracts based on 109 mtTR fragments from *P. cornutus. ***“QD” indicates the samples from Qingdao of Yellow Sea, and “DS” from Dongshan of East China Sea. The first digit indicates sample number while the bracketed digit is clone number.

A total of 109 CR fragments, including complete repeat tracts, were obtained by PCR amplification and cloning from 20 *P. cornutus* individuals (GenBank accession nos. JX457484-JX457592). The results show that all 109 CR fragments have repeat tracts with two or three motif types at their 3′ ends. The three motif types are designated: 5′-TR, M-TR, and 3′-TR based on their relative position in the CRs, with the 5′-TR near CSB3, the 3′-TR near *tRNA*^*Phe*^, and the M-TR between the 5′-TR and 3′-TR (Figure 1). The 5′- and 3′-TR motifs were detected in all 20 individuals while only two individuals (QD-07 and DS-03) contained the M-TR repeat. The motif sequence of the 5′-TR is ATATTACA and that of the M-TR motifs are shown in Table 1. The motif sequence of the 3′-TR is TTTAATGT; those 3′-TR motifs that varied by a single base are 3′-TR-v1 (T*G*TAATGT) and 3′-TR-v2 (T*A*TAATGT; Figures 1 and 2).

**Table 1:** **The sequences of M-TR motif in 20 individuals of *P. cornutus***

**Individuals**	**Sequences**
18 specimens	TTTATAACACTATTTATCAAAATACTCAAATTT GTGGTGCCCAGGATATTTAGAACACTTTAATG*
DS-03	(**TTTAA**AACACTATTTATCAAAATACTCAAATTT GTGGTGCCCAGGATATTTAGAACAC)_2_**TTTAA**TG
QD-07	TTTGT(**AACACT**ATTTATCAAAATACTCAAATTT GTGGTGCCCAGGATATTTAG)_4,5_**AACACT**TTAATG

*The sequences in 18 specimens are not repeated. Bold font: The bases at the 5′end of the repeat and bases identifying the 3′ flanking region.

### Hypervariable characteristics of 3′-terminal repeats

Comparisons of 109 3′-TRs showed that the length and the arrangement of 3′-TR and 3′-TR-v differ considerably not only between individuals, but also in different clones from single individuals (Figure 2). Extensive length polymorphism in these tracts is caused by differing motif copy number, ranging from four (DS-04, 32 bp) to 51 copies (QD-07, 408 bp) between individuals, and from four (DS-04, 32 bp) to 26 copies (DS-04, 208 bp) within individuals (Figure 2). A variable number of 3′-TR-v motifs are scattered throughout the 3′-TR tracts. The intra-individual differences are as follows: (i) One motif (3′-TR-v1) in DS-05(4), and two motifs (3′-TR-v1 and 3′-TR) in DS-11(3) are inserted. (ii) From one to six motifs are deleted at DS-13(6), QD-06(100), QD-07. (iii) While we can be sure that the 39^th^ copy of the motif is deleted in QD-07(8, 16, and 174), site of deletion within other clones cannot be easily identified. For example, a deletion of the 3′-TR-v2 in QD-07(6) could have occurred at any site from the 5^th^ to the 29^th^ copy of the motif (see Figure 2).

The variation of copy number and motif organization between individuals QD-02, QD-03, and QD-04 is negligible as the difference of copy number is no more than one copy. TR diversity is seen between individuals, particularly in comparison to QD-07, as the motif organization and repeat length in this individual are entirely different, and the sequence of the 3′-TR-v1 motif T***G***TAATGT, is changed to 3′-TR-v2 (T***A***TAATGT). The motif organization within a TR in individuals from different locations is not correlated with geographic location, indicating that TR organization is not geographically partitioned. For example, the TR organization of individuals QD-02, 03, 04 and DS-06, and 10 differ by a few 3′-TR motifs, but the TR organization of most other individuals vary to a great extent (Figure 2)

We suggest that: (i) There is remarkable sequence heterogeneity of 3′-TR tracts not only between individuals but also within individuals. (ii) Indel events occur randomly rather than at fixed sites within TR tracts. (iii) The indel unit of mtTRs could be one or multiple copies, suggesting that the indel tempo of mtTRs can be greatly increased by indel events of multiple motifs. (iv) There are two single base variants of 3′-TR:3′-TR-v1 and 3′-TR-v2, and the arrangement of 3′-TR and 3′-TR-v is highly diverse.

### Hypervariable features cannot be explained by available models

These data suggest that the low frequency of recombination events could not lead to such high levels of mtTR variation in both organization and length, thus it is impossible to explain the hypervariable features of *P. cornutus* TRs by the recombination model alone. Although the SSM model could explain such frequent variation, this model more easily explains the formation of shorter simple motifs (microsatellites) than the formation of longer repeated motifs [12]. If SSM is responsible for the mtTR variability in *P. cornutus*, simple shorter motifs would appear in higher abundance. For example, the repeated motif of 3′-TR-v2 (TA)_2_ATGT would include simple repeats, such as (TA)_3, 4,…_ATGT rather than (TATAATGT)_2, 3,…_ alone. However, the fact is that no such simple repeats were found in any of the 109 TR fragments suggests that the SSM is not the most suitable model to account for the hypervariable features in *P. cornutus* mtTRs*.* Similarly, it seems the IEM model cannot explains our data as this mechanism only takes effect at the 5′ end of the CR, while we observed repeated tracts at the 3′ end of the CR in this flatfish. Another mitochondrial model, IIM, would yield identical repeated arrays in length and location from *tRNA*^*Phe*^ to the origin of heavy strand replication, and this feature is not seen in *P. cornutus* mtTRs*.* Furthermore, the probability of false initiation of mt-replication from *tRNA*^*Phe*^, a condition for the IIM model, is not sufficiently high to explain the extremely hypervariable features in *P. cornutus* mtTRs. The model described by Hayasaka et al. [21] is suitable for the formation of TRs surrounding the CSB region, not a feature of the mtTRs in *P. cornutus*.

Repeated tracts in the mt genomes of other species such as dogs, wolves, and halibut [5,31] share similar hypervariable features to those of *P. cornutus*. Thus far, no mechanistic model can explain the origin of the observed variation in these species.

### The characteristics of tandem repeats in other vertebrate mt genomes

In order to explore the characteristics of tandem repeats in other vertebrate mt genomes, we retrieved 1,726 complete vertebrate mt genomes from in five taxonomic groups (Pisces, Amphibia, Reptilia, Aves and Mammalia) from the NCBI mt genome database. This data set includes 2,111 repeated tracts from 853 mt genomes (Table 2 and Additional file 1: Table S1). The repeated tracts in each mt genome were located. A total of 1,760 repeat tracts (83.4%) are concentrated mainly in the control regions, ranging from 65.4% in Amphibians to 90.5% in Reptiles, including 929 TRs at the 3′ end and 591 TRs at the 5′ end (we were not able to determine the relative positions of the remaining 240 TRs). These TRs exhibit considerable variation in copy number and motif length, with the former ranging from 2 to 158 copies, and the latter from 1 bp to 478 bp. Unexpectedly, 16.6% of the TRs are located outside the control regions, either entirely or partially in coding regions (179 TRs), rRNA genes (45 TRs), tRNA genes (40 TRs) and inter-genic regions (112 TRs). The length of most TRs in coding regions is less than 50 bp and the copy number ranges from two to four. This is the first report of so many repeated arrays located outside the control region (Additional file 1: Table S1).

**Table 2:** Characteristics of repeated tracts based in vertebrate mt genomes from NCBI

**Categories**	**Species**	**Species with TRs**	**Total TRs**	**At CRs**	**CR/Total**	At 3′*	At 5′*	**3′/CR**
Pisces	932	313	610	490	80.33%	156	316	33.05%
Amphibia	110	86	240	157	65.42%	59	64	47.97%
Reptilia	172	126	526	476	90.49%	290	84	77.54%
Aves	155	98	229	197	86.03%	144	28	83.72%
Mammalia	357	230	506	440	86.96%	280	99	73.88%
Total	1726	853	2111	1760	83.37%	929	591	61.12%

* 3′ or 5′ means 3′ or 5′ end in the CRs.

**Figure 3:**
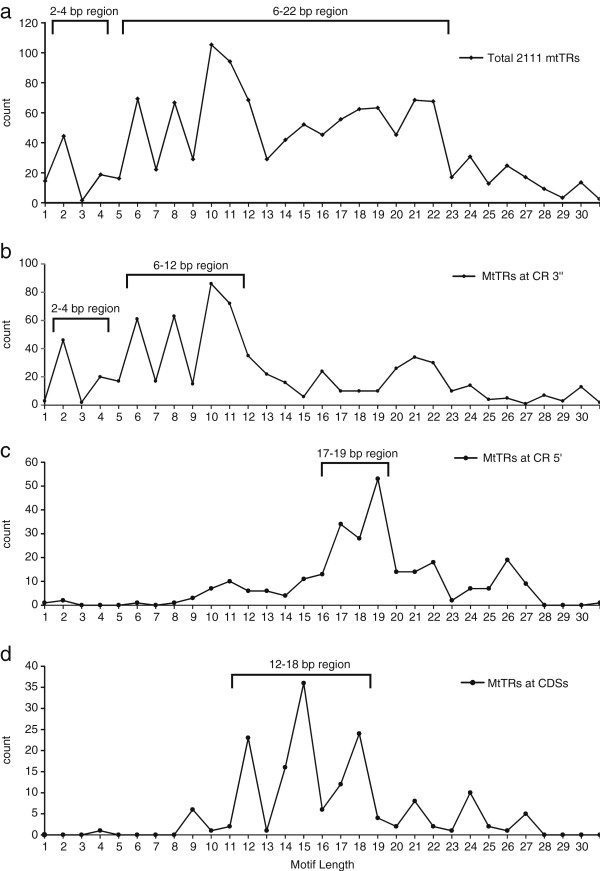
**The motif length distribution of mtTR tracts. a**. total 2111 mtTRs; **b**. mtTRs at the 3′ end in the CRs; **c**. mtTRs at the 5′ end in the CRs; **d**. mtTRs of CDSs.

The distribution of motif lengths of 2,111 mtTRs typically ranges between 6–22 bp (Figure 3a). The quantity of even-numbered motif lengths (2, 4, 6, 8 and 10 bp) is apparently more than those with odd numbers (3, 5, 7, 9 bp and 11 bp), a feature that has also been reported in nuclear genomes [32]. Because mtTRs are primarily located in the 3′ or 5′ ends of the CR and coding sequences, the motif length distributions at those locations were further analyzed, revealing length diversity (Figure 3b,c,d).

The TR motifs at the 3′ end are typically of two sizes: 2–4 bp and 6–12 bp (Figure 3b); those at 5′ end are typically much longer (17 to 19 bp; Figure 3c). Further analyses show that most motifs at the 5′ end (>60%) contain TAS blocks, which are not observed in TRs at the 3′ end. The motif length in coding sequences is usually 12–18 bp, and tends to vary in multiples of three bases (12, 15, 18; Figure 3d). It is assumed that a leading reason for the distribution of 17–19 bp motifs at the 5′ end is due to the selection pressure on TASs, because the TAS length is approximately 15 bp [33], while selection pressure to maintain reading frame leads to a three-base variation in coding sequences based on the triplet code.

It is generally believed that the indel of short motifs (microsatellites) can be explained by the SSM model [13,14]. The 1–4 bp repeats located at the CR 3′end (Figure 3b) may also be explained by this mechanism. There are, however, far more mtTRs with 6–22 bp motifs than those with shorter ones (Figure 3a). It is difficult to explain the generation of these larger motifs by the SSM model alone because the incidence of SSM decreases rapidly with increasing motif length. Additionally if long-motif mtTRs are generated by recombination, as happens in nuclear TRs [12], the low probability of mt recombination is not likely to generate the large number of mtTRs observed. Therefore, a new model of TR motif generation is needed.

### A novel model of Pause-Melting Misalignment (PMM)

Currently, no model can satisfactorily explain the variability of motif indels in mtTRs, particularly outside the CR region; the uneven distribution of motif sizes; and the concentration of mtTRs at both termini in the CR. A novel model of Pause Melting Misalignment (PMM) is proposed.

**Figure 4:**
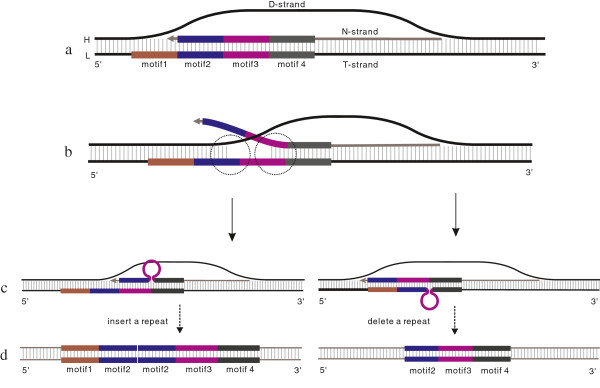
**The formation of a motif indel via the PMM mechanism. a**. the pause stage of mt-replication; **b**. competition and melting of the N-strand and D-strand; **c**. misalignment and protrusion; **d**. a repeated motif is inserted or deleted.

The process of PMM is activated by a pause event during mt-replication (Figure 4a). During this pause event, dynamic competition between the N-strand and the D-strand can lead to the melting of the N-strand from the T-strand (Figure 4b). As a result, the N-strand is partially displaced by the D-strand. When the N-strand successfully rebinds to the T-strand, mispairing between complementary or near-complementary sequences would occur easily due to possible single strand folding (Figure 4c). When next round of mt-replication or repair takes place, insertion or deletion of one or several motifs could occur (Figure 4d). If the self-complementary protrusion resides in the N-strand (Figure 4c), after the next round of mt-duplication, a repeat segment would be added (Figure 4d). If the protrusion resides in the T-strand (Figure 4c), the N-strand would delete a repeat segment after the next round of mt-replication (Figure 4d).

### The pause event of mitochondrial replication

During mt-replication, the pause event is the trigger for PMM. A pause event of mt-replication at the 5′ end in the CRs is likely since elongation of N-strands is arrested at TAS blocks at the 5′ end [33,34]. Similarly, we predict frequent pause events at the 3′ end in the CRs, because the replication initiation and closure domain of N-strands exist at this domain, many replication events such as segregation of N-strand molecules and superhelical turns [35] occur while the replication forks are traversing this section. These cases are likely to create frequent pause events in the replication process.

In addition to frequent replication pauses at the 5′ and 3′ ends, Koike and Wolstenholme [36] point out that mtDNA replication is a discontinuous process. Based on the observation of different molecular forms of mtDNA in electron micrographs, they found that at least 44% of the mt genome contains discrete positions at which DNA synthesis tends to be arrested. By using a combination of one- and two-dimensional agarose gel electrophoresis and solution hybridization to strand-specific probes, Mayhook et al. [37] proved that replication pauses exist at ATP6/COIII gene positions. These mt-replication pauses could occur not only at the CR, but also at other positions including protein coding sequences, tRNAs, and rRNAs. These pause events could trigger the PMM process, and lead to the formation and motif indel of mtTRs.

### Dynamic competition and melting

**Figure 5:**
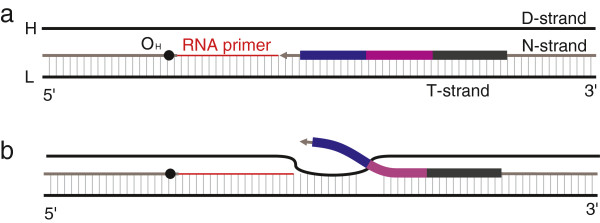
**The melting event at the 3′ end in the CR. a**. the D-strand is completely displaced at the 3′ end; **b**. the competitive state of the D-strand and the N-strand at 3′ end.

Buroker [18] reports that competitive equilibrium and melting occurs between the N-strand and D-strand at the TAS domains that could be followed by misalignment in repeated sequence in the white sturgeon mt genome. Because replication pause events exist at many sites during mt-replication, so too could the dynamic competition, double strand melting, and subsequent mispairing occur at multiple locations. These processes would provide the opportunity for the formation of repetitive motifs (Figure 4a,b,c). While mt-replication proceeds around the circular mt genome, the proportion of the D-strand binding to the T-strand gradually decreases from almost-entirely double stranded, to be completely displaced at the mt-replication termini (Figure 5a,b). Compared with the N-strand, the competitive strength of D-strand rebinding to the T-strand should therefore become much reduced, which would lead to a shorter melting length of the N-strand. This may be one of the reasons that the motif length at the 3′ end of the CR is generally shorter (Figure 3b,c).

### Folding and mispairing

Due to the competition of the D-strand, a portion of the N-strand is displaced from the T-strand, thus creating a short single-stranded status for both the N-strand and T-strand. These regions may then fold to form a stable intra-molecular secondary structure (Figure 4c), producing the shortened single strand of both strands, and then the reannealing of these regions could lead to mispairing. Induced stable secondary structures are thought to be a leading reason for mispairing during mtDNA replication [18,19], however, in our vertebrate mtTR data, the lengths of many motifs are 6, 8 bp or even 2–5 bp. Motifs of these lengths are not known to form stable secondary structures. Even if some motifs are known to form hairpin structures, such hairpins are usually unstable due to their high free energy (exceeding the criterion value, -0.6 kcal/mol). Thus, secondary structure formation does not appear to be necessary for the TR indel events.

**Figure 6:**
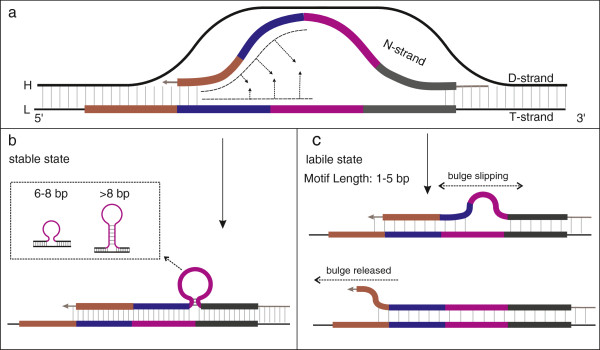
**The hypothesized process of mispairing and folding. a**. the 3′ end of the N-strand mispairing to the T-strand; **b**. the stable hairpin or loop protrusion; **c**. the labile state of the slipped bulge.

The inferred process for short motif mispairing and folding is as follows: The 3′ end of the N-strand mispairs with complementary sequences without any secondary structures formed (Figure 6a). The N-strand then gradually rebinds backwards with complementary bases until the unpaired bases are forced to form a hairpin, loop, or bulge, when the unpaired bases are longer than 8, from 6 to 8, or shorter than 6, respectively (Figure 6b,c)[38]. The bulge could slip backwards or forwards on the template strand in the labile state due to steric crowding of thymidine. If the bulge slips to the end of N-strand, it would be released (Figure 6c). The phenomenon of bulge slip may explain why motifs shorter than 6 bp (particularly 2–4 bp) are rarer than longer ones (Figure 3a). Another explanation may be that 2–4 nucleotides motifs cannot fold to any secondary structure, so the melted strands cannot be shortened, and the incidence of mispairing is decreased.

### The generation of M-TR motifs in *P. cornutus*

**Figure 7:**
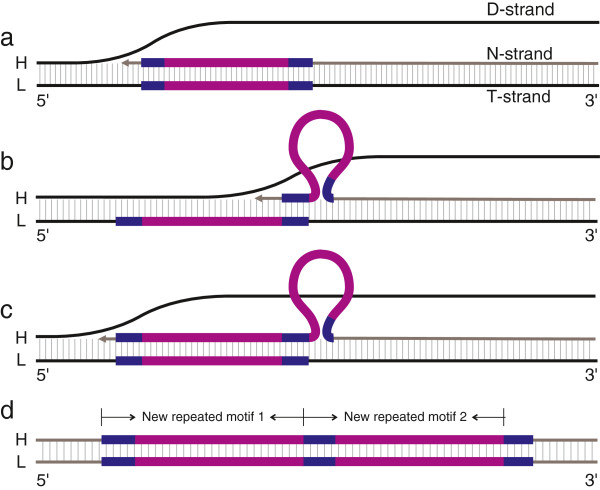
**Presumed process of M-TR motif formation in QD-07 and DS-03 of *P. cornutus*. a**. pause of mt-replication; **b**. misalignment and protrusion; **c**. restart and extending of the N-strand; **d**. new type motif is generated. The blue line represents the sequences AACACT in QD-07, TTTAA in DS-03; The red line represents the sequence ATTTATCAAAATACTCAAATTTGTGGTGCCCAGGATATTTAG in QD-07, and AACACTATTTATCAAAATACTCAAATTTGTGGTGCCCAGGATATTTAGAACAC in DS-03.

The mtTRs in *P. cornutus* are characterized by the presence of three motif types (3′, M, 5′-TR). Two types (3′, 5′-TR) were found in all 20 specimens, but the M-TR was only observed in DS-03 and QD-07 (Table 1). In three clones of DS-03, 58 of the 65 bases section is copied once and forms a new repeated tract; in 24 clones of QD-07, 48 of the 65 bases section is copied four (16 clones) or five times (8 clones). In the other 18 individuals, however, the same 65 bp section is not repeated (Table 1). These repeat tracts are indicative of the generation of new mtTRs. The new motifs share a feature in which the first several bases of the repeated motif are the same as the bases after the repeated motifs (bold sequences in Table 1). We suggest that the PMM model could be the mechanism by which the M-TR is formed (Figure 7). Specifically, after the melting of the N-strand due to dynamic competition, the 3′ end sequences of the N- strand inaccurately rebind (mispair) to complementary sequences of the T-strand. In the next round of mt-replication, a new repeat would be generated (Figure 7).

### The PMM model can readily explain key features of mtTRs in vertebrates

The PMM model differs from existing models of mtTR generation in several key aspects. Unlike the SSM model, the PMM model is specifically proposed for the origination and indel of motifs in mtTRs. It emphasizes the pause events as the trigger, followed by the melting of the N-strand from the T-strand, and subsequent folding and the mispairing. In contrast to the IEM and IIM models, the PMM model can explain the formation and indel of mtTRs in any locations of the mt genome. Furthermore, the PMM model can explain the origin of many characteristics of mtTRs in vertebrate mt genomes that the other models cannot.

In summary, the following characteristics of mtTRs can be explained by the PMM model: (i) The formation and motif indels of mtTR at any location in the mt genome can be produced by PMM mechanisms, even those outside the CR. Previous models do not address the production of indels at these locations. (ii) The concentration of large mtTRs at the 3′ and 5′ ends in the CRs is explained by the PMM model. Because there are stable pause events at both ends of the CR during mt-replication and the selection pressure upon the control region is less than that on other functional regions [1], additional TRs can be retained at these locations. (iii) Shorter motif lengths at the 3′ end could be due to reduced competition between the N-strand and the T-strand compared to that at the 5′ CR end. (iv) The generation of the M-TR motif in *P. cornutus* provides evidence for the origin of mtTRs by PMM mechanisms. (v) The PMM model can explain the hypervariable features of 3′-TRs in *P. cornutus*. Because mt-replication frequently pauses at the 3′ end of the CR, PMM would be triggered to generate a new copy or motif indels in existing mtTRs. The newborn motifs would subsequently increase the incidence of mispairing, which in turn would increase the frequency of motif indels. Furthermore, through the PMM model, more than one motif could be inserted or deleted in a single event, hence the generation tempo of motif indels greatly increases.

## Conclusions

MtTRs of *P. cornutus* exhibit hypervariable characteristics, thus the 3′-TR-v motifs could act as molecular markers for the exploration of the newly generated of mtTRs as well as future indels. Based on analyses of the mtTRs in *P. cornutus* and vertebrate mt genomes, we developed a novel model of Pause Melting Misalignment for the formation and motif indel of tandem repeats in the mt genome. The model can explain the characters of TRs in available mt genomes, and we hope this model will provide an important basis for the explanation of repeated region evolution in mt genomes.

## Competing interests

The authors declare that they have no competing interests.

## Authors’ contributions

WS collected datasets, carried out experiments, and drafted the manuscript. XYK directed the whole research work and revised the manuscript. ZMW, HXC and EADS help to draft and revise the manuscript. SSY carried out partial experiments. All authors read and approved the final manuscript.

## Supplementary Material

Additional file 1: Table S1 Detailed information of 2,111 repeated tracts from 853 complete mt genomes sequences. Click here for file
